# F2C2: a fast tool for the computation of flux coupling in genome-scale metabolic networks

**DOI:** 10.1186/1471-2105-13-57

**Published:** 2012-04-23

**Authors:** Abdelhalim Larhlimi, Laszlo David, Joachim Selbig, Alexander Bockmayr

**Affiliations:** 1 Department of Bioinformatics, Institute for Biochemistry and Biology, University of Potsdam, Karl-Liebknecht-Str. 24-25, D-14476 Potsdam, Germany; 2 Max-Planck Institute for Molecular Plant Physiology, Am Mühlenberg 1, D-14476 Potsdam, Germany; 3 FB Mathematik und Informatik, Freie Universität Berlin, Arnimallee 6, D-14195 Berlin, Germany; 4 DFG-Research Center Matheon, Arnimallee 6, D-14195 Berlin, Germany; 5 Berlin Mathematical School (BMS), Arnimallee 6, D-14195 Berlin, Germany

## Abstract

**Background:**

Flux coupling analysis (FCA) has become a useful tool in the constraint-based analysis of genome-scale metabolic networks. FCA allows detecting dependencies between reaction fluxes of metabolic networks at steady-state. On the one hand, this can help in the curation of reconstructed metabolic networks by verifying whether the coupling between reactions is in agreement with the experimental findings. On the other hand, FCA can aid in defining intervention strategies to knock out target reactions.

**Results:**

We present a new method F2C2 for FCA, which is orders of magnitude faster than previous approaches. As a consequence, FCA of genome-scale metabolic networks can now be performed in a routine manner.

**Conclusions:**

We propose F2C2 as a fast tool for the computation of flux coupling in genome-scale metabolic networks. F2C2 is freely available for non-commercial use at https://sourceforge.net/projects/f2c2/files/.

## Background

The huge amount of genomic, transcriptomic and related data has allowed for a fast reconstruction of an increasing number of genome-scale metabolic networks, e.g. [1-7]. In the absence of detailed kinetic information, constraint-based modeling and analysis has recently attracted ample interest due to its ability to analyze genome-scale metabolic networks using very few information [8-10]. Constraint-based analysis is based on the application of a series of constraints that govern the operation of a metabolic network at steady state. This includes the stoichiometric and thermodynamic constraints, which limit the range of possible behaviors of the metabolic network, corresponding to different metabolic phenotypes. Applying these constraints leads to the definition of the solution space, called the *steady-state flux cone*[11]:

(1) $$C=\{v\in R^{n}|\mathrm{Sv}=0,\;v_{i}\geq 0,\;\text{for all}\;i\in \mathrm{Irr}\},$$

where *S* is the *m*×*n* stoichiometric matrix of the network, with *m* internal metabolites (rows) and *n* reactions (columns), and the vector $v\in R^{n}$ gives a *flux distribution*. Furthermore, *Irr*⊆{1,…,*n*} denotes the set of *irreversible* reactions in the network, and *Rev*={1,…,*n*}∖*Irr* denotes the set of *reversible* reactions.

The flux cone contains the full range of achievable behaviors of the metabolic network at steady state. Various approaches have been proposed either to search for single optimal behaviors using optimization-based methods [12-16] or to assess the whole capabilities of a metabolic network by means of network-based pathway analysis [11,17-20].

Flux coupling analysis (FCA) is concerned with describing dependencies between reactions [21]. The stoichiometric and thermodynamic constraints not only determine all possible steady-state flux distributions over a network, they also induce coupling relations between the reactions. For instance, some reactions may be unable to carry flux under steady-state conditions. If a non-zero flux through a reaction in steady-state implies a non-zero flux through another reaction, then the two reactions are said to be coupled (see Def. 2 for a formal definition). FCA has been used for exploring various biological questions such as network evolution [22-24], gene essentiality [22], gene regulation [25-27], analysis of experimentally measured fluxes [28,29], or implications of the structure of the human metabolic network for disease co-occurrences [30]. Having a time efficient implementation of FCA is important in such studies.

After introducing the main existing algorithms for flux coupling analysis, we propose in this paper a new algorithm which significantly speeds up the calculation of flux coupling. Our algorithm is based on two main principles. First, we reduce the stoichiometric model as much as possible when parsing the stoichiometric matrix. Second, we use inference rules to minimize the number of linear programming problems that have to be solved. We prove the efficiency of our algorithm by successfully competing with the most recent approach [31]. We show that FCA can now be quickly performed even for very large genome-scale metabolic networks.

### Approaches for flux coupling analysis

Several algorithms were developed to calculate flux coupling between reactions. For a comparison among the existing approaches, the reader may refer to [31,32]. In the following, we focus on flux coupling methods based on solving a sequence of linear programming (LP) problems. These methods have proved to be significantly faster than other algorithms.

#### Definitions

We give a short overview of the important concepts we will use throughout this paper. First, we formally define blocked reactions in a metabolic network.

##### Definition 1 (Blocked reaction)

Given the steady-state flux cone *C*, let *i*∈{1,…,*n*} be a reaction. If *v*_*i*_=0, for all *v*∈*C*, reaction *i* is called blocked, otherwise *i* is unblocked.

In the following, we assume that the flux cone is not trivial, i.e., not all reactions are blocked. Next, we define the (un)coupling relationships between reactions.

##### Definition 2 (Coupling relations)

Let *i,j* be two unblocked reactions. The (un)coupling relationships $\overset{=0}{\to },\overset{=0}{↔},∽$ and ↦ are defined in the following way:

•$i\overset{=0}{\to }j$ if for all *v*∈*C*, *v*_*i*_=0 implies *v*_*j*_=0.

• $i\overset{=0}{↔}j$ if for all *v*∈*C*, *v*_*i*_=0 is equivalent to *v*_*j*_=0.

• *i*∽*j* if there exists *λ*≠0 such that for all *v*∈*C*,*v*_*j*_=*λv*_*i*_.

• *i * ↦ * j* if there exists *v*∈*C* such that *v*_*i*_=0 and *v*_*j*_≠0.

Reactions *i* and *j* are fully (resp. partially, directionally) coupled if the relation *i*∽*j* (resp. $i\overset{=0}{↔}j$, $i\overset{=0}{\to }j$) holds. Otherwise, *i* and *j* are uncoupled.

Note that *i*∽*j* (resp. $i\overset{=0}{↔}j$) is equivalent to *j*∽*i* (resp. $j\overset{=0}{↔}i$). In addition, *i*∽*j* implies $i\overset{=0}{↔}j$, which in turn is equivalent to $(i\overset{=0}{\to }j$ and $j\overset{=0}{\to }i)$.

As shown in [33] the reversibility type of reactions is a key concept in flux coupling analysis.

##### Definition 3 (Reversibility types)

A reversible reaction *i*∈*Rev* is called fully reversible if there exists a flux vector *v*∈*C* such that *v*_*i*_≠0 and *v*_*j*_=0 for all *j*∈*Irr*. Otherwise, reaction *i* is called pseudo-irreversible.

Using the reversibility type of reactions, we define the following reaction sets which will be used to determine the cases where flux coupling can occur between reactions.

•*Frev*={*i*∣*i* is fully reversible},

• *Prev*={*i*∣*i* is pseudo-irreversible and there exist *v*^ + ^,*v*^−^∈*C* such that $v_{i}^{+}>0,v_{i}^{-}<0\}$,

• *Irev*={*i*∣*i*∉*Frev*∪*Prev* and *v*_*i*_≠0 for some *v*∈*C*},

• *Blk*={*i*∣*i* is blocked }.

Note that the above reaction sets are disjoint and their union is equal to the set of all reactions, i.e., *Blk* ∪ *Irev* ∪ *Prev* ∪ *Frev*={1,…,*n*}.

As the classification of reactions according to their reversibility types has been treated elsewhere [31,33,34], we focus on calculating the flux coupling between reactions given the reaction sets *Blk*, *Irev*, *Prev* and *Frev*. First, we briefly review the main existing flux coupling methods based on linear programming. Afterwards, we present our new method to speed up the flux coupling analysis.

#### Flux coupling finder

The *Flux Coupling Finder (FCF)* algorithm [21] determines blocked reactions as well as coupled reactions by solving a sequence of linear programming (LP) problems. The FCF algorithm requires that each reversible reaction is split into two irreversible reactions, one forward and one backward. This may hamper the application of FCF to determine flux coupling in large genome-scale metabolic networks. Indeed, splitting reversible reactions results in an increase in the number of variables (resp. constraints) by |*Rev*| (resp. 2|*Rev*|). Since the FCF algorithm solves four LP problems to identify the maximum and minimum flux ratios for every pair of reactions, the total number of LP problems that have to be solved is very large. Furthermore, the FCF algorithm does not compute directly coupling relationships between reactions. A post-processing step is needed to deduce couplings between reactions (in the original network) from those between reaction directions (in the reconfigured network). More importantly, the FCF algorithm explores exhaustively all possible reaction pairs. This leads to a very big number of LP problems that have to be solved. This strategy may not scale well for genome-scale models of complex microorganisms which involve a large number of reactions.

#### Reversibility-based flux coupling analysis

To cope with the drawbacks of the FCF algorithm, we used the reversibility type of reactions to develop an improved version (WRP-FCF) of the original FCF method [31,34]. When looking for coupled reactions, WRP-FCF applies linear programming only in those cases where coupling relationships can occur [33]. Namely, given two unblocked reactions *i* and *j*, we have exactly three cases where a flux coupling is possible between *i* and *j*:

### Flux coupling between reversible reactions

If *i*,*j*∈*Prev* or *i*,*j*∈*Frev*, $i\overset{=0}{\to }j,i\overset{=0}{↔}j$ and *i*∽*j* are equivalent. More importantly, only the stoichiometric constraints determine whether $i\overset{=0}{\to }j$ holds, independently of the thermodynamic constraints.

Assume that blocked reactions have been identified beforehand and their respective columns in the stoichiometric matrix have been removed. Consider the following LP problem

(2) $$\max\{v_{j}\;\text{:}\;\mathrm{Sv}=0,\;v_{i}=0,\;0\leq v_{j}\leq 1\},$$

and let *v*^∗^ be an optimal solution. According to Proposition 6.13 in [34], $i\overset{=0}{\to }j$ if and only if $v_{j}^{*}=0$.

### Flux coupling between (pseudo-)irreversible reactions

If *i*∈*Irev* and *j*∈*Prev*, we only have to check whether $i\overset{=0}{\to }j$. The other coupling relationships cannot occur. Let *v*^1^ resp. *v*^2^ be optimal solutions of the two LP problems

(3) $$\begin{array}{l} \text{max}\{v_{j}\;\text{:}\;\mathrm{Sv}=0,\;v_{k}\geq 0,\;k\in \;\mathrm{Irr},\;v_{i}=0,\;v_{j}\leq 1\},\\ \text{min}\{v_{j}\;\text{:}\;\mathrm{Sv}=0,\;v_{k}\geq 0,\;k\in \;\mathrm{Irr},\;v_{i}=0,\;v_{j}\geq -1\}. \end{array}$$

Then $i\overset{=0}{\to }j$ if and only if $v_{j}^{1}=v_{j}^{2}=0$.

### Flux coupling between irreversible reactions

If *i*,*j*∈ *Irev*, in analogy with the FCF algorithm, we determine upper and lower bounds *U*_*ij*_ and *L*_*ij*_ such that 0≤*L*_*ij*_*v*_*j*_≤*v*_*i*_≤*U*_*ij*_*v*_*j*_ for all *v*∈*C* by solving the LP problems

(4) $$\begin{array}{l} L_{\mathrm{ij}}=\text{min}\;\{v_{i}\text{:}\;\mathrm{Sv}=0,\;v_{j}=1,\;v_{k}\geq 0,\;k\in \;\mathrm{Irr}\},\\ U_{\mathrm{ij}}=\text{max}\;\{v_{i}\text{:}\;\mathrm{Sv}=0,\;v_{j}=1,\;v_{k}\geq 0,\;k\in \;\mathrm{Irr}\}. \end{array}$$

Comparison of *L*_*ij*_ and *U*_*ij*_ allows us to determine whether reactions *i* and *j* are coupled using the following rules:

$i\overset{=0}{\to }j$ (resp. $j\overset{=0}{\to }i$) if and only if *L*_*ij*_≠0 (resp. *U*_*ij*_≠ + *∞*),

*j*∽*i* if and only if *L*_*ij*_≠0, *U*_*ij*_≠0 and *L*_*ij*_=*U*_*ij*_.

The improved version WRP-FCF does not make an exhaustive computation for all pairs of reactions and does not require a reconfiguration of the metabolic network. Accordingly, not only is the number of LP problems used by WRP-FCF smaller than the number solved by FCF, but also the LP problems used by WRP-FCF are simpler than those employed by FCF. For mathematical proofs, the reader may refer to [31,34].

#### Feasibility-based flux coupling analysis

Linear programming can be solved by enumerating a set of adjacent feasible solutions such that at each solution the objective function improves or is unchanged. Accordingly, searching for a feasible solution can be faster than computing an optimal solution [35]. Based on this observation, [31] proposed the FFCA approach for flux coupling analysis transforming the optimization-based LP problems used in the WRP-FCF method into feasibility-based LP problems. The authors compared FFCA with other available flux coupling algorithms, and showed that FFCA is slightly faster than WRP-FCF.

## Methods

Before using linear programming to calculate flux coupling between reactions, we preprocess the metabolic network in order to i) reduce the number of variables and constraints of the subsequent LP problems and ii) classify reactions according to their reversibility type. The network reduction is mainly based on the removal of trivially blocked reactions and the merging of the stoichiometric columns corresponding to trivially coupled reactions [36-39]. For this, one can use the kernel of the stoichiometric matrix. Alternatively, we can apply the following reduction rules which require a simple parsing of the stoichiometric matrix and are not time consuming. This strategy allows avoiding potential numerical instabilities related to the computation of a basis of the kernel.

### Preprocessing

**Figure 1:**
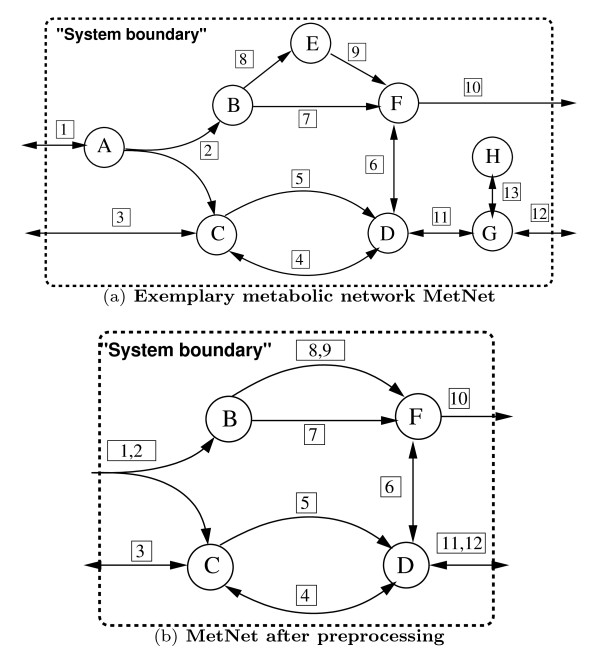
**Exemplary metabolic network MetNet before and after preprocessing.** (**a**) MetNet consists of eight metabolites (*A*,…,*H*), and thirteen reactions (1,…,13), whereof six reactions are irreversible. Metabolites are depicted as nodes while reactions are depicted as arrows. Reversible reactions are indicated by double arrowheads. (**b**) MetNet after preprocessing where dead-end metabolites and blocked reactions were removed and fully coupled reactions were merged iteratively. This resulted in the removal of the blocked reaction 13 and the merging of the pairs of equivalent reactions (1,2), (8,9) and (11,12)).

Certain metabolites, called *dead-end metabolites*[37], are produced (resp. consumed) by some reactions without being consumed (resp. produced) by other reactions. This concept is illustrated in Figure 1(a) where the dead-end metabolite *H* is produced by reaction 13 without being consumed by any of the remaining reactions.

As stated below, reactions which are consuming or producing dead-end metabolites are blocked.

#### Observation 1 (Dead-end Metabolite)

Let *k*∈{1,…,*m*} be a metabolite. Then, the following hold:

If there exists a reaction *i* such that *S*_*ki*_≠0 and *S*_*kj*_=0 for all *j*≠*i*, then reaction *i* is blocked.

If there exists a set of reactions *I*⊆*Irr* such that *S*_*ki*_>0 (resp. *S*_*ki*_<0) for all *i*∈*I* and *S*_*kj*_=0 for all *j*∉*I*, then all reactions *i*∈*I* are blocked.

In each of these cases, *k* is called a dead-end metabolite.

Certain metabolites are involved in exactly two reactions. For instance, in the network MetNet depicted in Figure 1(a), metabolite *E* is produced/consumed only by reactions 8 and 9. The following observation states that the fluxes through reactions involving such metabolites are proportional to each other.

#### Observation 2 (Trivial Full Coupling (TFC))

Let *i* and *j* be two reactions such that, for some metabolite *k*∈{1,…,*m*}, *S*_*ki*_≠0, *S*_*kj*_≠0 and *S*_*kl*_=0 for all *l*≠*i*,*j*. Then, reactions *i* and *j* are either blocked or fully coupled.

The identification of dead-end metabolites and their corresponding blocked reactions allows us to reduce the number of metabolites and reactions that matter for identifying coupled reactions. As stated in the following observation, the removal of the rows (resp. columns) in the stoichiometric matrix corresponding to dead-end metabolites (resp. blocked reactions) does not influence the flux coupling between reactions.

#### Observation 3 (Reduction Rule 1)

Let *D* be a set of dead-end metabolites and let *B* be a set of blocked reactions. For convenience, suppose *B*={*s* + 1,…,*n*}. Let *S*^*′*^ be the submatrix of *S* formed by the rows *S*_*k*∗_ with *k*∉*D* and the columns *S*_∗*l*_ with *l*∉*B*. Let *Irr*^*′*^=*Irr*∖*B*. Then, for all pairs of reactions *i*∉*B* and *j*∉*B*,

•$i\overset{=0}{\to }j$ if and only if $v_{i}^{\prime }=0$ implies $v_{j}^{\prime }=0$, for all $v^{\prime }\in R^{s}$ such that *S*^*′*^*v*^*′*^=0 and $v_{p}^{\prime }\geq 0\;\text{for all}\;p\in {\mathrm{Irr}}^{\prime }$.

• *i*∽*j* if and only if there exists *λ*^*′*^≠0 such that $v_{j}^{\prime }=\lambda^{\prime }v_{i}^{\prime }$, for all $v^{\prime }\in R^{s}$ with *S*^*′*^*v*^*′*^=0 and $v_{p}^{\prime }\geq 0\;\text{for all}\;p\in {\mathrm{Irr}}^{\prime }$.

The next observation shows that two fully coupled reactions can be represented by only one column in the stoichiometric matrix, without altering the flux coupling between reactions.

#### Observation 4 (Reduction Rule 2)

Let *k*,*l* be two reactions such that for all *v*∈*C*,*v*_*l*_=*λv*_*k*_ for some *λ*≠0. For convenience, suppose *l*=*n* and *λ*>0. Let *S*^*′*^ be the *m*×(*n*−1) matrix defined by $S_{*p}^{\prime }=S_{*p}$ for all *p*≠*k*,*l* and $S_{*k}^{\prime }=S_{*k}+\lambda S_{*l}$. Let *Irr*^*′*^=(*Irr*∪{*k*})∖{*l*} if *l*∈*Irr*, and *Irr*^*′*^=*Irr* otherwise. Then, for all pairs of reactions *i*≠*l* and *j*≠*l*,

$i\overset{=0}{\to }j$ if and only if $v_{i}^{\prime }=0$ implies $v_{j}^{\prime }=0$, for all $v^{\prime }\in R^{n-1}$ such that *S*^*′*^*v*^*′*^=0 and $v_{p}^{\prime }\geq 0\;\text{for all}\;p\in {\mathrm{Irr}}^{\prime }$.

*i*∽*j* if and only if there exists *λ*^*′*^≠0 such that $v_{j}^{\prime }=\lambda^{\prime }v_{i}^{\prime }$, for all $v^{\prime }\in R^{n-1}$ with *S*^*′*^*v*^*′*^=0 and $v_{p}^{\prime }\geq 0\;\text{for all}\;p\in {\mathrm{Irr}}^{\prime }$.

Note that when applying the reduction rules of Observations 3 and 4, further metabolites and reactions may fulfill the conditions of Observations 1 and 2. Accordingly, we apply these reduction rules in an iterative way. As an illustration, the reduction of the network MetNet depicted in Figure 1(a) involves two iterations. In the first one, metabolite *H* and reaction 13 are removed, the pairs of reactions (1,2) and (8,9) are merged and metabolites *A* and *E* are removed. In the second iteration, the equivalent reactions (11,12) are merged and metabolite *G* is removed. The preprocessed network depicted in Figure 1(b) contains only four metabolites and nine reactions.

Certain fully coupled reactions could not be identified using Observation 2. The following lemma proves that all fully coupled reaction pairs can be deduced from the kernel $\mathrm{kern}(S)=\{v\in R^{n}|\mathrm{Sv}=0\}$ of the stoichiometric matrix after the removal of all blocked reactions.

#### Lemma 1

Let (*S*,*Irr*) be a metabolic network with *n* unblocked reactions. For a pair of reactions (*i*,*j*) the following are equivalent:

*i*∽*j*.

there exists λ∈R∖{0} such that *v*_*i*_=*λv*_*j*_, for all *v*∈*kern*(*S*).

#### Proof

⇐" Immediate.

"⇒" Since *i*∽*j*, there exists *λ*≠0 such that *v*_*i*_=*λv*_*j*_ for all *v*∈*C*. Assume by contradiction that there is *v*∈*kern*(*S*) such that *v*_*i*_≠*λv*_*j*_ and let *L*={*l*∈*Irr*∣*v*_*l*_<0}. Since every reaction is unblocked, for every *l*∈*L* there exists *g*^(*l*)^∈*C* with $\left(g_{l}^{\left(l\right)}=1\right)$. Let $w=v-\underset{l\in L}{\sum }v_{l}g^{\left(l\right)}$. Clearly, *w*∈*kern*(*S*) and *w*_*l*_>0 for all *l*∈*Irr*, thus *w*∈*C*. However, *w*_*i*_≠*λw*_*j*_, contradicting *i*∽*j*. The required statement follows. □

In analogy with the WRP-FCF and FFCA approaches, we identify the reversibility type of reactions in order to apply linear programming only in those cases where coupling relationships can occur [33]. Here, we use the procedure for reaction classification described in [31,34]. Note that applying the above reduction rules beforehand reduces the number of variables and constraints in the LP problems used for the classification of reactions.

Based on the results above, we propose to apply the preprocessing procedure given in Table 1 before identifying coupled reactions using linear programming. We show later that the preprocessing step turns out to be crucial for obtaining an efficient flux coupling algorithm.

**Table 1:** Main steps of the preprocessing procedure

**Step**	**Rule**
1.	Identify dead-end metabolites and the corresponding blocked reactions.
2.	Apply Reduction Rule 1 to remove the rows (resp. columns) corresponding to dead-end metabolites (resp. blocked reactions) from the stoichiometric matrix.
3.	Apply the TFC rule to determine reactions which are proportional to each other and update their reversibility type.
4.	Apply Reduction Rule 2 to keep only one column for each set of reactions which are proportional to each other.
5.	Repeat Steps 1-4 until neither a dead-end metabolite nor a pair of fully coupled reactions can be identified.
6.	Update the reversibility type of each reaction and remove the columns corresponding to blocked reactions from the stoichiometric matrix.
7.	Use a basis of the kernel of the stoichiometric matrix to identify fully coupled reactions. This step is optional as the kernel computation may lead to numerical problems.
8.	Classify reactions according to their reversibility type.

### Algorithmic improvements

In certain metabolic networks, the conversion of a set of substrates into a set of products can be made by different reactions having the same stoichiometry. A simple example of such reactions are isoenzymes which make the same conversion of substrates into products. This concept is illustrated in Figure 1(a) where both reactions 4 and 5 convert *C* into *D* in the same way. This holds also for reaction 7 and the merged equivalent reactions (8,9) in Figure 1(b) showing that the network preprocessing may simplify the identification of such alternative conversions. The flux coupling of such reactions is trivial using the following lemma.

#### Lemma 2 (Trivial Uncoupling (TUC))

Let *i*,*j*∈*Irev* and *k*,*l*∈*Prev*∪*Frev* be four reactions. Then, the following holds:

• If *S*_∗*i*_=*αS*_∗*j*_ for some *α*>0, then *i*↦*p* and *j*↦*p* for all *p*∉*Blk*.

• If *S*_∗*i*_=*αS*_∗*j*_ for some *α*<0, then *p*↦*i* (resp. *p*↦*j*) for all *p*∉*Blk*∪{*j*} (resp. *p*∉*Blk*∪{*i*}).

• If *S*_∗*i*_=*αS*_∗*k*_ for some *α*≠0, then *i*↦*p* and *p*↦*i* for all *p*∉*Blk* and *q*↦*k* for all *q*∉*Blk*∪{*i*}.

• If *S*_∗*k*_=*αS*_∗*l*_ for some *α*≠0, then *k*↦*p* and *p*↦*k* for all *p*∉*Blk*∪{*l*} and *l*↦*q* and *q*↦*l* for all *q*∉*Blk*∪{*k*}.

#### Proof

The proofs of the four statements are similar. We only consider the first one. Suppose *S*_∗*i*_=*αS*_∗*j*_ for some *α*>0 and let us prove *i*↦*p*. Let *p*∉*Blk*. There exists *v*∈*C* such that *v*_*p*_≠0. Let $w\in R^{n}$ such that *w*_*i*_=0,*w*_*j*_=*αv*_*i*_ + *v*_*j*_ and *w*_*q*_=*v*_*q*_ for all *q*≠*i*,*j*. We have *w*∈*C*,*w*_*i*_=0,*w*_*p*_≠0 and so the claim follows. □

The next observation states that metabolites which are involved only in irreversible reactions and consumed or produced by exactly one reaction define trivial directional couplings between these reactions.

#### Observation 5 (Trivial Directional Coupling (TDC))

Let *k* be some metabolite such that *S*_*kl*_=0 for all *l*∈*Frev*∪*Prev*. Let *P*={*i*∣*S*_*ki*_>0} and *N*={*j*∣*S*_*kj*_<0}. If *card*(*P*)=1 (resp. *card*(*N*)=1), then $i\overset{=0}{\to }j$ (resp. $j\overset{=0}{\to }i$) for all (*i*,*j*)∈*P*×*N*.

Since directional flux coupling is a transitive relation, the flux (un)coupling between many reactions can simply be deduced from dependencies between reactions whose flux (un)coupling has been determined beforehand. This allows us to significantly reduce the total number of LP problems to be solved. Examples of such inferred flux (un)couplings are given in Figure 1(b). According to the TDC rule, we have $\left(1,2)\overset{=0}{\to }(8,9\right)$. By solving the LP problems (4), we obtain 10↦(8,9). We can easily conclude that 10↦(1,2).

Table 2 shows the inferred flux (un)coupling relations we can deduce from known relationships between reactions.

**Table 2:** Transitivity inferred flux (un)coupling

**Known flux coupling**	***i***∽***j***	$i\overset{=0}{↔}j$	$i\overset{=0}{\to }j$	$j\overset{=0}{\to }i$
*k*∽*i*	*k*∽*j*	$i\overset{=0}{↔}j$	$k\overset{=0}{\to }j$	$j\overset{=0}{\to }k$
		*k*∽*j*		
$k\overset{=0}{↔}i$	$k\overset{=0}{↔}j$	$k\overset{=0}{↔}j$	$k\overset{=0}{\to }j$	$j\overset{=0}{\to }k$
$k\overset{=0}{\to }i$	$k\overset{=0}{\to }j$	$k\overset{=0}{\to }j$	$k\overset{=0}{\to }j$	
*k*↦*i*	*k*↦*j*	*k*↦*j*		*k*↦*j*
*i*↦*k*	*j*↦*k*	*j*↦*k*	*j*↦*k*	

For some pairs of reactions, we need to solve at least one LP problem. The optimal solution not only determines the flux coupling between the considered pair of reactions, but also allows one to determine many other uncoupled reactions.

#### Observation 6 (Feasibility Rule)

Let *v*∈*C* be a steady state flux vector and let *I*={*i*∣*v*_*i*_=0} and *J*={*j*∣*v*_*j*_≠0}. Then *i*↦*j* for all (*i*,*j*)∈*I*×*J*.

In general, most irreversible reactions are uncoupled to each other. Accordingly, the LP problems (4) used to determine coupled irreversible reactions are often unbounded. This limits the use of the feasibility rule, which requires the calculation of a feasible flux vector. In order to optimally use the feasibility rule, instead of solving the LP problems (4) to decide whether two irreversible reactions *i*,*j*∈*Irev* are coupled, we propose to solve the bounded LP problems

(5) $$\begin{array}{cc} L_{\mathrm{ij}} & =\text{min}\{v_{i}\;\text{:}\;\mathrm{Sv}=0,\;v_{j}=1,\;v_{k}\geq 0,\;k\in \mathrm{Irr}\},\\ L_{\mathrm{ji}} & =\text{min}\{v_{j}\;\text{:}\;\mathrm{Sv}=0,\;v_{i}=1,\;v_{k}\geq 0,\;k\in \mathrm{Irr}\}, \end{array}$$

and to use the following scheme:

$i\overset{=0}{\to }j$ (resp. $j\overset{=0}{\to }i$) if and only if *L*_*ij*_≠0 (resp. *L*_*ji*_≠0),

*j*∽*i* if and only if *L*_*ij*_≠0, *L*_*ji*_≠0 and *L*_*ij*_=1/*L*_*ji*_.

The following observation states that removing a fully reversible reaction does not alter the flux coupling between (pseudo-)irreversible reactions.

#### Observation 7

Let *k*∈*Frev* be a fully reversible reaction. For convenience, suppose *k*=*n*. Let *S*^*′*^ be the *m*×(*n*−1) matrix defined by $S_{*p}^{\prime }=S_{*p}$ for all *p*≠*k* and let *Irr*^*′*^=*Irr*. Then, for all pairs of reactions *i*∉*Frev* and *j*∉*Frev*,

$i\overset{=0}{\to }j$ if and only if $v_{i}^{\prime }=0$ implies $v_{j}^{\prime }=0$, for all $v^{\prime }\in R^{n-1}$ with *S*^*′*^*v*^*′*^=0 and $v_{p}^{\prime }\geq 0\;\text{for all}\;p\in {\mathrm{Irr}}^{\prime }$.

*i*∽*j* if and only if there exists *λ*^*′*^≠0 such that $v_{j}^{\prime }=\lambda^{\prime }v_{i}^{\prime }$, for all $v^{\prime }\in R^{n-1}$ with *S*^*′*^*v*^*′*^=0 and $v_{p}^{\prime }\geq 0\;\text{for all}\;p\in {\mathrm{Irr}}^{\prime }$.

Let *S*_∗*Rev*_ be the submatrix defined by the columns in *S* corresponding to the reversible reactions and let *t* be the dimension of *kern*(*S*_∗*Rev*_). Based on Observation 7, we can remove up to *t* independent fully reversible reactions without altering the flux coupling between (pseudo-)irreversible reactions. Since certain fully reversible reactions may change their reversibility type after the deletion of a fully reversible reaction, we first remove a randomly chosen reaction *i*∈*Frev* together with the coupled reactions with *i*. We calculate the impact of this deletion on the dimension of *kern*(*S*_∗*Rev*_). If this dimension decreases by 1, the deletion is maintained; otherwise the removed reactions are restored to the network. This is repeated until *t* independent fully reversible reactions and their coupled reactions are removed. We assume that the flux coupling between fully reversible reactions is determined beforehand.

Based on the above results, we propose the Fast Flux Coupling Calculator (F2C2) to determine coupled reactions. The main steps of the F2C2 algorithm are given in Table 3.

**Table 3:** The main steps of the F2C2 algorithm

**Step**	**Rule**
1.	Apply the preprocessing procedure shown in Table 1.
2.	Apply the feasibility rule using the feasible solutions obtained when solving the LP problems used in Step 1.
3.	Apply the TDC and TUC rules to determine trivially (un)coupled reactions.
4.	Identify fully coupled reversible reactions by solving the LP problems (2). This is not necessary if the kernel of the stoichiometric matrix is used in Step 1.
5.	Determine the dimension *t* of *kern*(*S*_∗*Rev*_) and remove *t* independent fully reversible reactions and their coupled reactions. This step is optional since *t* is often small.
6.	Determine the flux coupling between (pseudo-)irreversible reactions by solving the LP problems (3) and (5).
7.	For each LP problem solved in Step 6, use the inference rules in Table 2 in combination with the feasibility rule.

## Results and discussion

The F2C2 algorithm has been implemented within the *MATLAB* environment, using *CLP* (via the Mexclp [40] interface) as the underlying linear programming solver. For benchmarking, we analyzed the following genome-scale metabolic networks: *Escherichia coli*, *iJR*904 [1], *Saccharomyces cerevisiae*, *iND*750 [2], *Methanosarcina barkeri*, *iAF*692 [3], *Mycobacterium tuberculosis*, *iNJ*661 [4], *Escherichia coli*, *iAF*1260 [5], *Homo sapiens*, *Recon* 1 [6] and *Escherichia coli*, *iJO*1366 [7]. For the numerically sensitive parts, a tolerance level of 10e-6 was set. All computations were performed using a single Intel Xeon 5160 (3.0 GHz) processor on a 64-bit Debian Linux 6.0 system.

As pointed out in the previous section, part of the performance gain of F2C2 over previous FCA algorithms stems from the fact that the preprocessing steps reduce the network size. This affects the running time on two levels: there are fewer reaction pairs and the LP problems to be solved have reduced size. The dramatic effect of the preprocessing steps on the network size is presented in Table 4.

**Table 4:** **Genome-scale metabolic networks with the number of metabolites (*♯*met.) and reactions (*♯*reac.) before and after applying the preprocessing steps**

	**Original size**		**Prepr. size**	
**Network name**	***♯*met.**	***♯*reac.**	***♯*met.**	***♯*reac.**
*M. barkeri*, *iAF*692	628	690	149	221
*S. cerevisiae*, *iND*750	1061	1266	248	446
*M. tuberculosis*, *iNJ*661	826	1025	240	418
*E. coli*, *iJR*904	761	1075	269	565
*E. coli*, *iAF*1260	1668	2382	604	1272
*E. coli*, *iJO*1366	1805	2582	651	1376
*H. sapiens*, *Recon*1	2766	3742	725	1573

The algorithmic improvements further reduce the number of LP problems to be solved. A direct performance comparison between the FFCA and F2C2 algorithms (including the running times and number of LP problems solved) is summarized in Table 5. In all cases, F2C2 outperformed FFCA by several orders of magnitude. In [31] it has been shown that FFCA is more efficient on genome-scale metabolic networks than other flux coupling algorithms.

**Table 5:** **Performance comparison between the FFCA and F2C2 algorithms in terms of the number of LP problems solved (*♯*LPs) and their total running times (TRT)**

	**FFCA**		**F2C2**		
**Network**	***♯*LPs**	**TRT**	***♯*LPs**	**TRT**	**Prepr. RT**
*M. barkeri*, *iAF*692	301975	59 m 40 s	774	7 s	5 s
*S. cerevisiae*, *iND*750	472629	1 h 50 m 17s	1280	21 s	15 s
*M. tuberculosis*, *iNJ*661	556504	3 h 5 m 36 s	1506	22 s	16 s
*E. coli*, *iJR*904	655437	2 h 40 m 33 s	1580	26 s	18 s
*E. coli*, *iAF*1260	4256786	4 d 31 m 26 s	3309	2 m 47 s	2 m
*E. coli*, *iJO*1366	4877262	4 d 5 h 30 m 46 s	3955	3 m 55 s	2 m 45 s
*H. sapiens*, *Recon*1	4566304	4 d 18 h 3 m 37 s	3903	5 m 20 s	4 m 9 s

For the F2C2 algorithm, TRT includes the time (Prepr. RT) required for the preprocessing step. Computation times are given in days (d), hours (h), minutes (m) and seconds (s).

**Figure 2:**
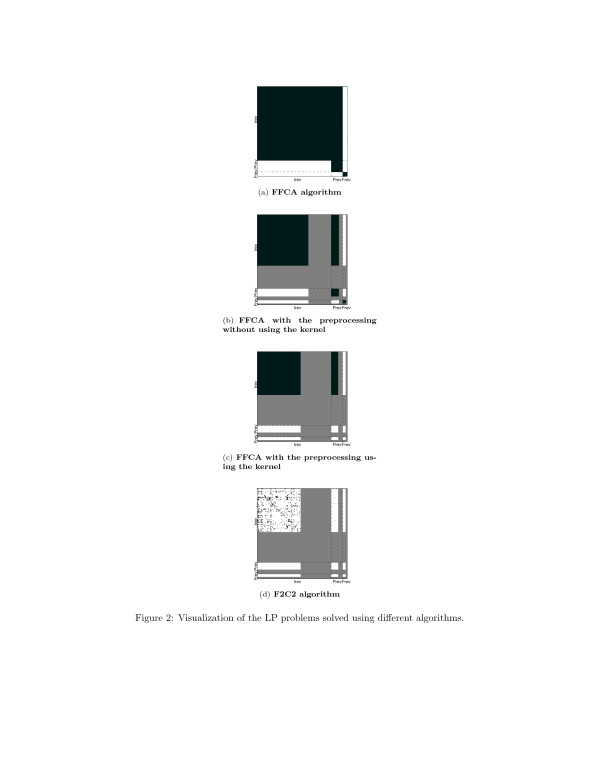
**Visualization of the LP problems solved using different algorithms.** (**a**) The FFCA algorithm, (**b**) the FFCA algorithm after applying the preprocessing procedure given in Table 1 without kernel computation (Step 7), (**c**) the FFCA algorithm after applying the preprocessing procedure and using the kernel of the stoichiometric matrix to identify fully coupled reactions and (**d**) the F2C2 algorithm given in Table 3. The dashed lines mark the boundary of the *Irev*, *Prev* and *Frev* regions. Colors: Black - an LP problem is solved for the corresponding (ordered) pair of reactions; Gray - the corresponding LP problem is not solved due to one (or both) reactions being eliminated from the network (a preprocessing improvement); White - corresponding LP problem does not get solved due to an algorithmic improvement.

For an intuitive, visual representation of the individual improvements’ impact on the algorithm’s performance, a more in-depth analysis has been performed on the recent metabolic network of *E. coli, iJO*1366. Four different sets of improvements were cumulatively switched on, and the linear programs solved were plotted for each case (Figure 2). To better highlight the relevant differences, the following modifications were applied to the results. First, 249 (out of 2582) reactions identified as blocked were removed from the images. This is a common preprocessing step in most FCA algorithms. Secondly, the order of reactions was permuted such that the reactions in *Irev*, *Prev* and *Frev* are clustered together. Additionally, in each of these three clusters, the fully coupled reactions were moved towards the end of the segment.

Figure 2(a) plots the LP problems solved in the FFCA algorithm. Applying the simple preprocessing steps without using the kernel (Figure 2(b)), several reactions are found to be fully coupled with others, and as such can be merged together. When Lemma 1 is applied (Figure 2(c)), all fully coupled sets are detected. As a consequence the gray stripes get thicker and the LP problems corresponding to (*Prev, Prev*) and (*Frev, Frev*) pairs need not be solved anymore. The use of the algorithmic improvements (Figure 2(d)) filters the pairs in (*Irev, Irev*) and (*Irev, Prev*) blocks, greatly reducing the total number of LP problems solved.

## Conclusions

We have presented the new flux coupling algorithm F2C2, which outperforms previous methods by orders of magnitude. Flux coupling analysis of genome-scale metabolic networks can now be performed in a routine manner. A software tool F2C2 is freely available for non-commercial use at https://sourceforge.net/projects/f2c2/files/.

## Abbreviations

F2C2: Fast Flux Coupling Calculator; FCA: Flux Coupling Analysis; FFCA: Feasibility-based Flux Coupling Analysis; FCF: Flux Coupling Finder; LP: Linear Program; TDC: Trivial Directional Coupling; TFC: Trivial Full Coupling.

## Competing interests

The authors declare that they have no competing interests.

## Authors’ contributions

The F2C2 algorithm was designed by all authors, based on ideas from AL and LD. The implementation and computational experiments were done by LD. The first draft of the manuscript was written by AL, and revised by LD and AB. The final document was approved by all authors.
